# Role of Preoperative Echocardiography in Resource-Limited Settings and Delayed Patient Care

**DOI:** 10.7759/cureus.105936

**Published:** 2026-03-26

**Authors:** Zamanali Khakhar, Shamshuddin Mohammedali, Sayed K Ali

**Affiliations:** 1 Medicine, University of Nairobi, Nairobi, KEN; 2 Orthopedic Surgery, Aga Khan University Hospital, Nairobi, KEN; 3 Medicine, Aga Khan University Hospital, Nairobi, KEN

**Keywords:** echocardiography, femur and fracture, less is more, perioperative echo, pre operative evaluation

## Abstract

Preoperative echocardiography has long been used as an effective diagnostic tool in the preoperative assessment of surgical candidates. In elderly patients, hip fractures represent a critical medical emergency with a high risk of complications that can occur if not addressed promptly. Unnecessary preoperative investigations may lead to delays and potentially compromise the quality of patient care. The motivation to evaluate the utility of preoperative echocardiography in select clinical scenarios, particularly in low- and middle-income countries (LMICs) where resources are limited, arose from the case of a 72-year-old female who presented to a tertiary hospital in Nairobi, Kenya, with a right-sided intertrochanteric hip fracture following a traumatic fall. She was physically active with metabolic equivalents of tasks (METs) ≥4 and had no significant history of cardiovascular symptoms or risk factors. Preoperative electrocardiogram (ECG) was performed and revealed T-wave inversions in leads V5-V6, prompting a request for an echocardiogram. Technical difficulties led to a delay in the procedure, postponing surgery. The patient was later deemed fit for surgery by the cardiology team without the need for an echocardiogram, and she successfully underwent an open reduction and internal fixation (ORIF).

Routine preoperative echocardiography serves minimal clinical benefit in asymptomatic and stable patients undergoing non-cardiac surgery. In such patients, metabolic equivalents of tasks (METs ≥4), normal N-terminal pro-B-type natriuretic peptide (NT-proBNP), and cardiac troponin levels can serve as sufficiently reliable tools for preoperative cardiovascular evaluation. Unnecessary echocardiograms have been associated with surgical delays, prolonged hospital stays, and increased healthcare costs. Evidence-based medical practices guided by established guidelines are essential in ensuring efficient patient care. In addition to obtaining a detailed patient history and thorough physical examination, it is also crucial to inform clinicians of the selective yet valuable role of echocardiograms in the preoperative assessments of surgical patients to prevent futile interventions and delays.

## Editorial

Echocardiography serves as a useful diagnostic modality for assessing cardiac structure and function, especially in patients who might lead a sedentary lifestyle. Its relative ease of use and ability to be portable, compared to other cardiovascular imaging techniques, are distinctly favorable, especially for patients undergoing surgical intervention [1]. Hip fractures represent a critical medical emergency, particularly among elderly patients, with a high risk of complications if not addressed promptly. Current guidelines recommend performing surgical repair of a hip fracture within 24 hours of admission to optimize postoperative functional outcomes and reduce mortality, hospital stay, and complications [2]. However, the routine use of preoperative procedures, such as echocardiograms, without clear indications may inadvertently undermine these objectives.

The motivation to evaluate the utility of preoperative echocardiography in select clinical scenarios, particularly in low- and middle-income countries (LMICs) where resources are limited, arose from the case of a 72-year-old woman with a remote history of breast cancer in remission who presented after a mechanical fall, sustaining a right intertrochanteric hip fracture. Prior to her fall, she maintained an active lifestyle and was able to walk for 45 minutes four times a week with metabolic equivalents of tasks (METs) ≥4 and was independent in activities of daily living and had no history of cardiovascular disease, diabetes, or kidney disease. Laboratory investigations were within normal limits, including renal and thyroid panels, glycated hemoglobin (HbA1c) (6.05%), troponin, and N-terminal pro-B-type natriuretic peptide (NT-proBNP).

Her admission electrocardiogram (ECG) showed T-wave inversions in V5-V6 without prior tracings for comparison; serum troponin and pro-B-type natriuretic peptide (NT-proBNP) levels were within normal ranges, and the patient was asymptomatic with no prior history of cardiovascular disease. Although the anesthesia team requested a transthoracic echocardiogram, the procedure was aborted due to technical challenges, as the patient was unable to reposition onto her left side. This resulted in a 48-hour delay in the planned surgical intervention, after which surgery was performed.

Cardiology review the following day deemed her fit for surgery without formal echocardiography. She underwent open reduction and internal fixation 48 hours after admission using a cephalomedullary nail. The procedure was performed under spinal epidural anesthesia with a fascia iliaca block, and the patient was classified as American Society of Anesthesiologists (ASA) physical status classification system class I. There were no immediate postoperative complications observed. She mobilized the next day and was discharged 24 hours postoperatively.

Echocardiographic assessment is frequently performed as a routine standard procedure without clear clinical indications, potentially leading to superfluous and unnecessary testing, delay in patient care, and resource misallocation. The latter is of particular concern in resource-limited settings, such as LMICs, where healthcare resources are scarce. There is a need to evaluate the appropriateness of preoperative echocardiography in the context of established guidelines for preoperative cardiovascular assessment. We set out to understand its clinical utility in various situations in which other interventions may take precedence over routine echocardiographic evaluation. In our setting of LMICs, where access to preoperative echocardiography may be limited, alternative approaches to cardiovascular risk stratification may ensure safe and effective perioperative care.

The AHA/American College of Cardiology (ACC) and multiple other authoritative bodies concur that in asymptomatic, clinically stable patients undergoing non-cardiac surgery (NCS), preoperative evaluation of the left and right ventricular function via echocardiograms provides no clinical benefit and hence is not recommended [3]. Furthermore, the American Board of Internal Medicine (ABIM) Foundation, in collaboration with the American Society of Echocardiography (ASE), as part of the Choosing Wisely® campaign, advocates for the rational use of echocardiograms. They recommend against routine echocardiograms for preoperative or perioperative assessment of patients with no history of cardiovascular symptoms [4]. A cohort study involving 2,298 surgical candidates who underwent echocardiography following a hip fracture revealed that preoperative echocardiography was associated with increased postoperative morbidity and mortality. A plausible explanation for the observed increase in mortality is that the use of preoperative echocardiography may contribute to delays in the timing of surgical intervention. Moreover, preoperative echocardiograms were associated with prolonged surgical delays, extended hospital stays, and higher total healthcare costs. However, further research is required to reliably establish a causal relationship between preoperative echocardiography and postoperative mortality [5]. 

Echocardiograms can be of added value if used in the appropriate clinical setting. Patients with new onset or worsening dyspnea, physical exam findings consistent with heart failure, or altered ventricular function or unexplained symptoms in the setting of an elevated NT-proBNP would certainly benefit from an echocardiogram to aid in patient risk stratification and optimizing care prior to surgery [3,4]. 

Functional capacity remains a reliable predictor for adverse cardiovascular events after NCS. It is generally assessed in METs. Typically, METs less than 4 reflect poor functional capacity, whereas METs of at least 4 indicate acceptable functional capacity [4]. A MET exceeding 4 suggests that the patient can climb a flight of stairs or walk uphill. In contrast, an inability to walk more than two blocks on level ground or run a short distance indicates a MET below 4, reflecting poor functional capacity and a higher risk of postoperative cardiac events [6].

Stable patients with an exercise capacity of ≥4 METs, as in the case presented here, have a reduced risk of perioperative cardiac events and, in most cases, do not require any additional cardiovascular testing. Use of both NT-proBNP and cardiac troponin may serve as a reasonable tool in patients >65 years of age undergoing elevated-risk NCS. The aforementioned professional bodies also suggest that an abnormal preoperative ECG might not alter the perioperative management, except in cases with significant abnormalities such as second-degree Mobitz type II or higher atrioventricular block, atrial fibrillation with rapid ventricular response, new-onset atrial fibrillation, or a prolonged QT interval [3].

According to the 2024 AHA/ACC perioperative cardiovascular management guidelines, routine preoperative echocardiography is not recommended in asymptomatic and clinically stable patients undergoing NCS. There is insufficient evidence to support routine preoperative assessment of left ventricular function in stable patients. Large studies have demonstrated no improvement in survival, outcomes, or shorter hospital stays associated with preoperative echocardiography. A review of existing clinical guidelines suggested that, according to current recommendations, further evaluation is needed only in patients presenting with clinical heart failure, significant arrhythmias, unstable coronary syndromes, or severe valvular disease [7].

Echocardiographic evaluation should be reserved for patients with new or worsening cardiac symptoms or when findings are expected to influence perioperative management [3]. An important consideration is the HIP ATTACK trial. It provides valuable context in the evaluation of preoperative investigations in patients undergoing hip fracture surgery. A randomized controlled trial was carried out to assess a strategy of accelerated time to surgery compared with standard care. While accelerated assessment and surgery did not significantly reduce mortality or major perioperative complications, it was associated with a lower risk of delirium, reduced pain, and suggested improvements in other patient-centered outcomes [8]. In higher-risk patients, decisions regarding preoperative investigations are more complex and nuanced, necessitating a more detailed and selective approach to their assessment. 

In the presented case, the postponement of the surgical procedure for over 48 hours was attributed to delays in completion of an unnecessary preoperative echocardiogram in a physically active patient (METs ≥4) with no cardiovascular risk or symptoms, which ultimately resulted in a prolonged hospital stay and increased costs. The optimal allocation of echocardiography as a preoperative diagnostic tool is influenced by resource availability in resource-limited settings and the financial constraints of patients requiring this evaluation. Consequently, the implementation of evidence-based practices is crucial to enhancing the efficiency of patient care, particularly in LMICs, where healthcare resources are often limited. In resource-limited settings, the limited availability of echocardiography may represent a considerable burden, particularly in remote healthcare facilities and underfunded institutions. In LMICs, where many patients often face significant financial constraints, routine echocardiography may impose an additional economic burden on those already struggling to access basic healthcare services. Consequently, its indiscriminate use as a routine preoperative procedure in patients without clear indications may delay timely care while also unnecessarily increasing financial strain. Its selective and efficient use may therefore reduce surgical waiting times and improve patient outcomes.

Aligning diagnostic practices with current guidelines can potentially enhance patient experiences and outcomes, reduce delays in intervention, and minimize overall healthcare costs. In addition to obtaining a detailed patient history and conducting a thorough physical exam, it is also crucial to understand the selective role of echocardiograms in preoperative assessments of surgical candidates, thereby optimizing the quality of care for patients and preventing futile interventions and delays.
